# Pharmacological targeting of Tripartite Motif Containing 24 for the treatment of glioblastoma

**DOI:** 10.1186/s12967-021-03158-w

**Published:** 2021-12-09

**Authors:** Mingzhi Han, Yanfei Sun

**Affiliations:** Cheeloo College of Medicine, Shandong University, Jinan, 250012 China

**Keywords:** Glioblastoma, TRIM24, Cancer stem cells, Cell viability, Cell Invasion

## Abstract

Glioblastoma (GBM) is the most aggressive brain tumor of the central nervous system. Recent studies have reported the crucial functions of Tripartite Motif Containing 24 (TRIM24) in promoting cancer progression of GBM. However, it remains unclear if TRIM24 is an attractive druggable target for therapeutic intervention in GBM. We therefore performed a series of experiments, aiming to verify whether specific TRIM24 inhibition suppresses GBM malignant functions using dTRIM24 and IACS-9571, two novel selective TRIM24 antagonists. Our data showed that TRIM24 inhibitors serve as effective agents for inhibiting cell propagation and invasion of several patient-derived GBM stem cells (GSCs), and these effects are mediated partially through suppression of the TRIM24-SOX2 axis. This study provides novel insight into the TRIM24-based druggable dependencies, important for developing effective therapeutic strategies for brain tumors.

**Supplementary Information:**

The online version contains supplementary material available at 10.1186/s12967-021-03158-w.

**Letter to the editor**

Glioblastoma multiforme (GBM) is the most aggressive brain tumor of the central nervous system. The ability of tumor cells to migrate, rapidly diffuse and invade normal adjacent tissue, their sustained proliferation, and the existence of GBM stem cells (GSCs) leads to a median survival of approximately 15 months following the best standard of care [1–3]. Therefore, it is of paramount importance to understand the molecular mechanisms contributing to GBM development and progression to develop more effective therapies.

Tripartite Motif Containing 24 (TRIM24), also known as TIF1α, is an important member of the Transcription Intermediary Factor (TIF) family. It consists of a RING-type E3 ubiquitin ligase domain, and a terminal plant homeodomain (PHD)-bromodomain which acts as a reader of the non-canonical histone signature H3K23ac. TRIM24 has been shown to function as an oncogenic factor or tumor suppressor dependent on the cancer type. For instance, aberrant overexpression of TRIM24 is associated with oncogenesis and disease progression in a wide variety of cancers including breast cancer, gastric cancer, and GBM [4]. Recently, Zhang et al. [5] showed TRIM24 to be highly expressed in GSCs where the binding, through its bromodomain, activates the expression of the pluripotency transcription factor Sex-determining region Y–box 2 (SOX2), -thereby promoting GBM stemness and invasiveness. Through a TRIM24 shRNA knockdown approach and functional assays, it was suggested that TRIM24 represents a potential target for GBM treatment. Recently, potent and specific inhibitors for TRIM24 have been developed [6, 7]. It is therefore important to validate the translational significance of these findings in a pharmacological context.

To determine the antitumor effects of TRIM24-based druggable dependencies, we used two novel TRIM24 inhibitors: (i) IACS-9571, a high-affinity, potent dimethyl-benzimidazole bromodomain inhibitor of TRIM24/BRPF1 with good selectivity over other bromodomain family proteins without modifying TRIM24 expression level [6]; (ii) dTRIM24, a bifunctional degrader of TRIM24 based on proteolysis-targeted chimera (PROTAC). dTRIM24 can selectively bind both the bromodomain of TRIM24 and the E3 ubiquitin ligase VHL, thus driving proteasome-mediated degradation of TRIM24 [7]. We determined the effects of these two compounds on a panel of patient-derived GSC lines which have been well characterized (Additional file 1: Table S1). Both dTRIM24 and IACS-9571 effectively and dose-dependently reduced the proliferation of GSCs (Fig. 1A). Furthermore, the treatment of GSCs with dTRIM24 (5, 10 μM) or IACS-9571 (10, 20 μM) attenuated the capacity of tumorsphere formation (Fig. 1B and Additional file 1: Fig. S1) and the expression of stemness markers SOX2 and Nestin through immunofluorescence staining (Fig. 1C), demonstrating that pharmacological targeting of TRIM24 effectively inhibits self-renewal of GSCs. Moreover, western blot analysis showed a decrease in TRIM24 and SOX2 expression levels after dTRIM24 treatment (Fig. 1D, upper), verifying its efficacy as a TRIM24 protein degrader in GSCs. Likewise, treatment of IACS-9571 caused a decrease of SOX2 (Fig. 1D, lower). We further observed that both compounds attenuated the invasion distance of GSCs (P < 0.001; Fig. 1E) and induced cell apoptosis, while the cell cycle was not significantly affected (Additional file 1: Fig. S2). In a rescue experiment, ectopic expression of SOX2 in GBM#P3 cells partially restored cellular viability suppression followed by IACS-9571 or dTRIM24 treatment compared to the control group (Fig. 1F and Additional file 1: Fig. S3), suggesting that TRIM24-SOX2 axis was involved in the inhibitory effects of these two inhibitors. TRIM24 has been reported to contribute to GBM progression via several signaling pathways. For instance, Zhang et al. [8] found that TRIM24 could bind to the *PIK3CA* (Phosphoinositide-3-Kinase Catalytic Alpha Polypeptide) promoter, thus enhancing PI3K/Akt signaling in GBM cell lines. Lv et al. [4] showed that TRIM24 could cooperatively activate Signal Transducer and Activator of Transcription 3 (STAT3) signaling and enhance Epidermal Growth Factor Receptor (EGFR)-driven GBM tumorigenesis, indicating multifaceted roles of TRIM24 in the GBM signaling networks. Therefore, further multi-omics studies are warranted in order to elucidate the molecular mechanisms underlying the effects of the TRIM24 inhibitors in GBM.

**Fig. 1:**
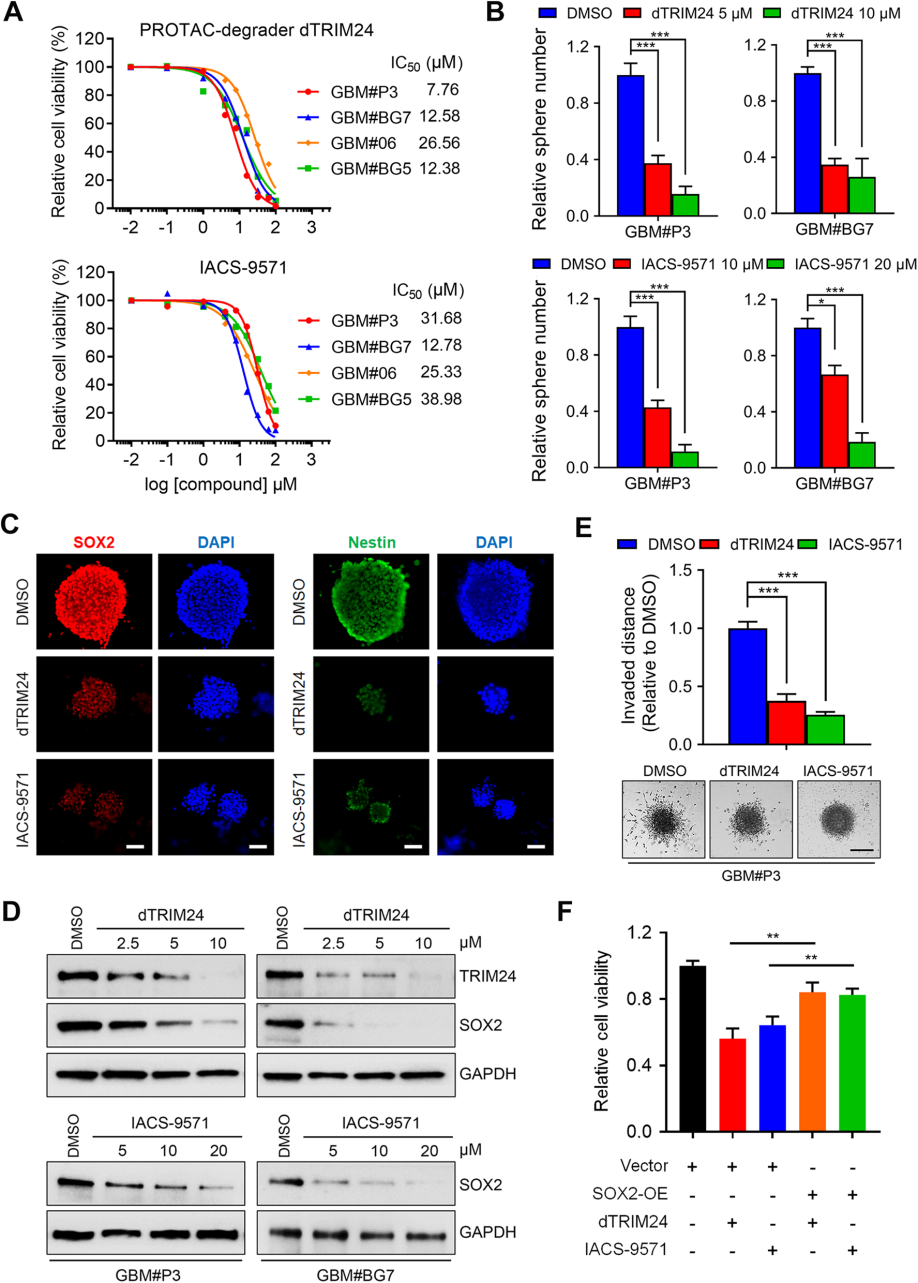
**A** IC_50_ curves for dTRIM24 (MedChemExpress, USA) and IACS-9571 (MCE, hydrochloride form) in GBM#P3, GBM#BG7, GBM#06, and GBM#BG5 cells using the Cell Titer-Glo viability assay. **B** Quantification of tumoursphere formation assays for GSCs treated with different concentrations of dTRIM24 (0–10 μM) (A) or IACS-9571 (0—20 μM) for 6 days. GSCs (1000 cells/mL/well) were seeded in 6-well ultra-low adhesion plates. Inverted phase-contrast microscopy was used to count the sphere number. **C** Representative images of immunofluorescence staining for SOX2 (red; dilution 1: 100) or Nestin (green; dilution 1: 200) in GBM#P3 treated with dTRIM24 or IACS-9571 for 48 h. Nuclei were counterstained with DAPI (blue). Scale bar = 100 μm. **D** Western blot analyses of the TRIM24, SOX2, and GAPDH in lysates (20 µg) from GSCs treated with different concentrations dTRIM24 (0–10 μM) or IACS-9571 (0–20 μM). **E** Representative images of spheroids in 3D invasion assays for GBM#P3 GSCs treated with DMSO, dTRIM24 (5 μM), or IACS-9571 (10 μM), and evaluated at 24 h. Scale bar = 200 μm (lower). Graphic representation of the quantification of the distance of invading cells from the tumorspheres determined after 24 h (upper). **F** Relative cell viability for rescue experiments using the Cell Titer-Glo viability assay in GBM#P3 cells as indicated. Data are shown as mean ± SEM. Statistical significance was determined by one‐way ANOVA. *P < 0.05, **P < 0.01, ***P < 0.001

## Conclusions

In conclusion, our data show for the first time that TRIM24 inhibitors serve as effective agents for targeting GSCs, and these inhibitory effects are partially mediated through suppression of the TRIM24-SOX2 axis. These observations, together with the reported ability of dTRIM24 and IACS-9571 to inhibit growth and trigger apoptosis in a panel of acute monocytic leukemia cells [7], make TRIM24 an attractive drug target for therapeutic intervention in GBM. It is also noteworthy that the dTRIM24 is more effective in displacing TRIM24 from chromatin compared to IACS-9571 and exerts a pronounced effect on TRIM24 target genes [7], which is consistent with our findings that dTRIM24 has a relatively lower IC_50_ in GSCs. Based on the Molinspiration Cheminformatics (http://www.molinspiration.com) prediction, the Topological Polar Surface Area (TPSA) score of IACS-9571 (TSPA: 122.5) and dTRIM24 (TSPA: 260.0) show that these compounds have relatively moderate to low values of blood–brain barrier (BBB) penetration. This implies that pharmacologically targeting TRIM24 for the treatment of GBM might not, at present, be achieved in a preclinical and clinical context. Yet, optimization of their chemical structures and new therapeutic developments toward TRIM24 warrant further exploitation.

## Supplementary Information

**Additional file 1.** Additional Figures S1–S3 and Table S1.

## Data Availability

All data generated or analyzed during this study are included in this article.

## Abbreviations

GBM: Glioblastoma
TRIM24: Tripartite Motif Containing 24
TIF: Transcription Intermediary Factor
PHD: Plant homeodomain
GSCs: GBM stem cells
PROTAC: Proteolysis-targeted chimera
SOX2: Sex-determining region Y–box 2
PIK3CA: Phosphoinositide-3-Kinase Catalytic Alpha Polypeptide
STAT3: Signal Transducer and Activator of Transcription 3
EGFR: Epidermal Growth Factor Receptor
