# Supplementary material for: Pharmacological targeting of Tripartite Motif Containing 24 for the treatment of glioblastoma
Source: J Transl Med. 2021 Dec 9;19:505. doi: 10.1186/s12967-021-03158-w (PMC8662887; doi:10.1186/s12967-021-03158-w)
Supplement: Supplementary file 1 — Additional file 1. Additional Figures S1–S3 and Table S1. [file 12967_2021_3158_MOESM1_ESM.pdf]

# Supplementary Materials for

## Pharmacological targeting of Tripartite Motif Containing 24 for the treatment of glioblastoma

Mingzhi Han

Correspondence to: mingzhi.han@sdu.edu.cn

Figures. S1 to S3
Tables S1

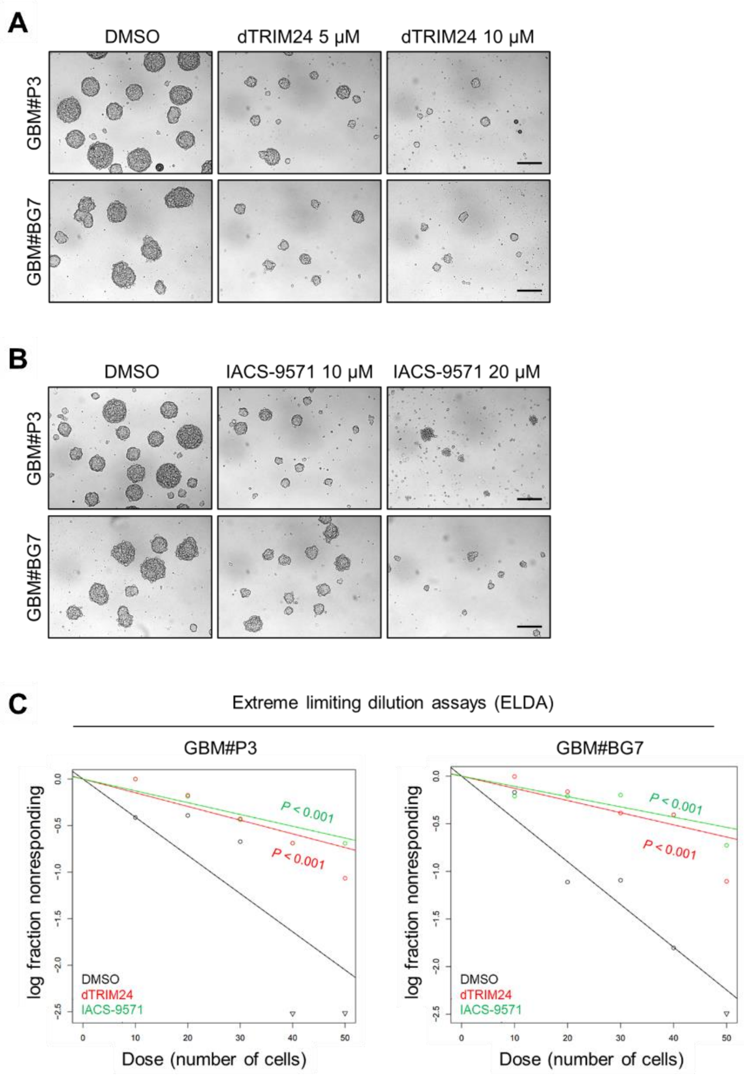


**Figure S1. (Related to Fig.1B)**. Graphic representation of tumoursphere formation assays for GSCs treated with different concentrations of dTRIM24 (0 - 10 µM) (A) or IACS-9571 (0 - 20 µM) (B) for 6 days. Scale bar = 500 µm. For the tumoursphere formation assay, GSCs single-cell suspensions (1000 cells/mL/well) were seeded in 6-well ultra-low adhesion plates (Corning Inc., USA) and cultured in Neurobasal medium supplements and growth factors. Inverted phase contrast microscopy (Nikon; Japan) was used to count and acquire images of the tumourspheres. (C) For the in vitro extreme limiting dilution assay, GSCs were placed in a 96-well plate at a density of 1 to 50 cells/well with six replicates for each concentration. After ten days, the numbers of tumourspheres in each well were determined, and the sphere formation efficacy was calculated using extreme limiting dilution analysis software

(http://bioinf.wehi.edu.au/software/elda/). Statistical significance was determined by one-way ANOVA. *P < 0.05, **P < 0.01, ***P < 0.001.

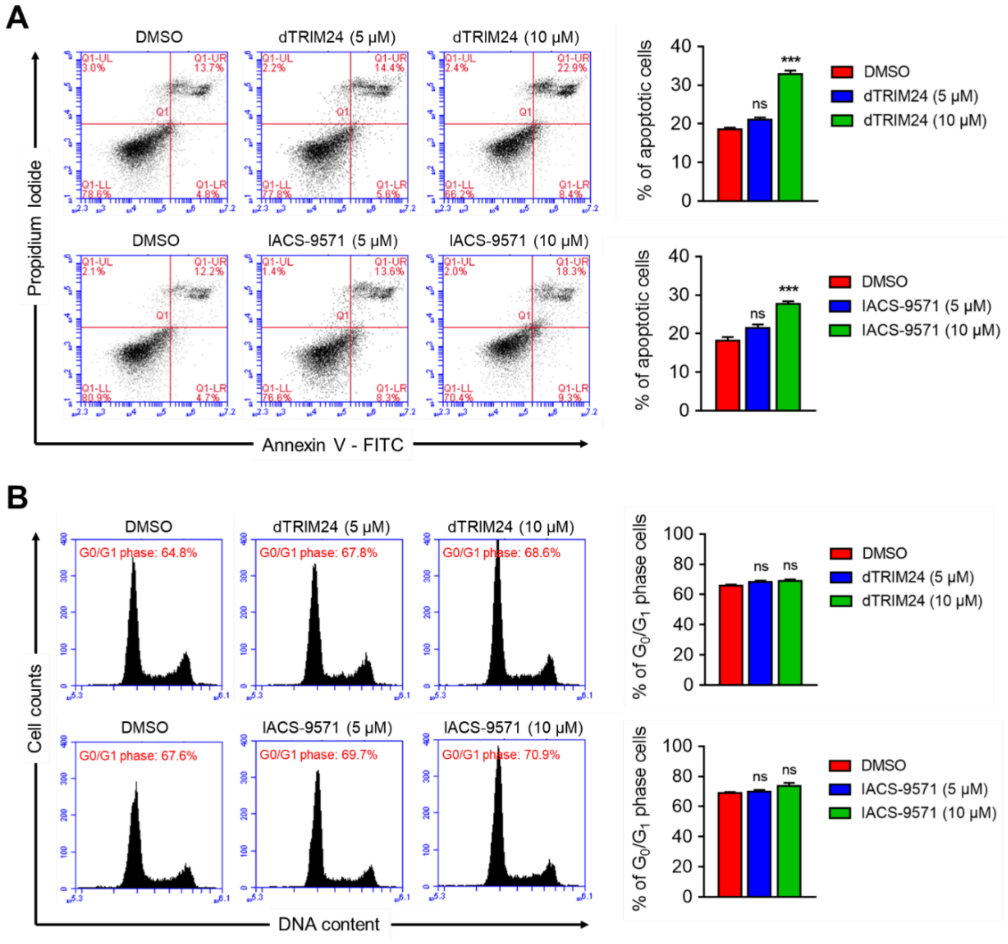


**Figure S2.** Flow cytometry and the quantification for the apoptosis assessment (A) and cell cycle analysis (B) in GBM#P3 GSCs treated with DMSO, dTRIM24, or IACS-9571, and evaluated after 48 h. For the cell cycle analysis, GSC cells were harvested, resuspended, and stained with propidium iodide (PI; BD Biosciences) in the presence of RNase A for 20 min, according to the manufacturer's protocol. Apoptosis was evaluated by Annexin V-FITC and PI staining (20 min). Cells were analyzed using a flow cytometer (BD Biosciences). Data were analyzed and presented using NovoExpress (ACEA Biosciences, USA). Statistical significance was determined by one-way ANOVA. *P < 0.05, **P < 0.01, ***P < 0.001.

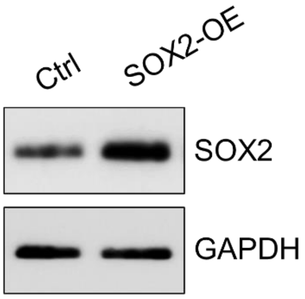


**Figure S3. (Related to Fig.1F)**. Overexpression of SOX2 (SOX2-OE) in GBM#P3 confirmed by western blot assay. GAPDH was used as an internal normalization control. The full-length ORF of the gene was cloned into pENTER vectors. Transfection was performed with Lipofectamine 3000 (Life Technologies, CA).

**Supplementary Table S1**. STR profile information of primary GBM cells used in this study.

| Sample No. GBM#P3 | |
|---|---|
| Marker | Allele |
| D19S433 | 16.2 |
| D5S818 | 10,11 |
| D21S11 | 31.2,33.2 |
| D18S51 | 12,15 |
| D6S1043 | 12,18 |
| AMEL | X,Y |
| D3S1358 | 14,16 |
| D13S317 | 11,12 |
| D7S820 | 14 |
| D16S539 | 11,12 |
| CSF1PO | 10,12 |
| Penta D | 10,11 |
| D2S441 | 11 |
| vWA | 16,17 |
| D8S1179 | 14,15 |
| TPOX | 8 |
| Penta E | 5,15 |
| TH01 | 9 |
| D12S391 | 19,26 |
| D2S1338 | 17,23 |
| FGA | 23,25 |

| Sample No：GBM#BG7 | |
|---|---|
| Marker | Allele |
| D3S1358 | 15,17 |
| TH01 | 9,9.3 |
| D21S11 | 31.2,32.2 |
| D18S51 | 12 |
| Penta E | 16,17 |
| D5S818 | 12 |
| D13S317 | 8 |
| D7S820 | 10 |
| D16S539 | 12,13 |
| CSF1PO | 11,13 |
| Penta D | 10,13 |
| AMEL | X |
| vWA | 14,19 |
| D8S1179 | 14 |
| TPOX | 8 |
| FGA | 21,22 |
| D19S433 | 12,13 |
| D12S391 | 20,21 |
| D6S1043 | 11,20 |
| D2S1338 | 17,20 |
| D1S1656 | 17.3 |

| Sample No：GBM#BG5 | |
|---|---|
| Marker | Allele |
| D3S1358 | 18,19 |
| TH01 | 9 |
| D21S11 | 29,32.2 |
| D18S51 | 13,18 |
| Penta E | 7,11 |
| D5S818 | 11,13 |
| D13S317 | 11,13 |
| D7S820 | 10 |
| D16S539 | 10,11 |
| CSF1PO | 10,11 |
| Penta D | 9,13 |
| AMEL | X |
| vWA | 15,16 |
| D8S1179 | 8,10 |
| TPOX | 8 |
| FGA | 19,21 |
| D19S433 | 14,15 |
| D12S391 | 23 |
| D6S1043 | 11,14 |
| D2S1338 | 17,21 |
| D1S1656 | 12 |

| Sample No：CBM#06 | |
|---|---|
| Marker | Allele |
| D3S1358 | 14,15 |
| TH01 | 6,9.3 |
| D21S11 | 28,29 |
| D18S51 | 16 |
| Penta E | 12 |
| D5S818 | 12,13 |
| D13S317 | 12 |
| D7S820 | 10,11 |
| D16S539 | 9,10 |
| CSF1PO | 11 |
| Penta D | 13,14 |
| AMEL | X,Y |
| vWA | 16 |
| D8S1179 | 13 |
| TPOX | 8,11 |
| FGA | 21 |
| D19S433 | 15 |
| D12S391 | 17.3 |
| D6S1043 | 12 |
| D2S1338 | 18 |
| D1S1656 | 11,12 |
